# Alternating Droplet Formation by using Tapered Channel Geometry

**DOI:** 10.1038/s41598-018-19966-y

**Published:** 2018-01-25

**Authors:** Muhammad Saqib, O. Berkay Şahinoğlu, E. Yegân Erdem

**Affiliations:** 1 Mechanical Engineering Department, Bilkent University, Ankara, 06800 Turkey; 2 UNAM (National Nanotechnology Research Center), Ankara, 06800 Turkey

## Abstract

The ability to produce a controlled sequence of alternating droplets from two separate sources inside a microfluidic system brings several advantages in microfluidic analysis. The effectiveness of this technique for use in an application depends on the ability of the device to replicate the pattern continuously and accurately. In this work we studied the effect of the dispersed phase channel geometry on generating a repeating pattern of alternating droplets in a cross junction microfluidic device. By measuring the radius of curvature of a droplet at the time of break up, and calculating the Laplace pressure using these values, we analyzed how the angle of taper of the dispersed phase inlet channel has an influence on the pattern repetition and uniformity of formed droplet size and spacing in between. The performance of devices with different angle of taper values were studied experimentally. This comparative study indicated that the ability of a cross junction device to generate alternating droplets with uniform size and spacing is highly dependent on the angle of taper of the inlet channels; and it improves with larger taper angles.

## Introduction

The research field of microfluidics has many attractive aspects like high throughput analysis, use of small quantity of reactants, addressing samples individually, precise control over reaction conditions and possibility of automation and integration^1,2^. One branch of microfluidics is the droplet-based microfluidics that allows controlled manipulation of reagents in microchannels in the form of individual packages –or droplets. This significantly improves the uniformity of conditions such as temperature and residence time among the samples carried in droplets due to chaotic advection^3^, which is not possible to achieve in non-droplet based (continuous) flow regimes due to laminar flow^4^. It also provides rapid mixing of samples within droplets. Moreover there is no contamination on channel walls in droplet based devices in contrast to continuous flow devices due to the thin film of continuous phase that is between droplets and channel walls^5^. Due to these advantages droplet based microfluidics has specifically found applications in biological analysis^6^, chemical synthesis^7^, high-throughput screening^8^ and other micro total analysis systems (µTAS)^9^.

In order to increase the advancement in the analysis and synthesis, it is important to have controlled droplet generation such that size and formation periods of droplets are known. In this paper, the droplet generation geometry is discussed to form alternating droplet sequence by using two different sources. This pattern generation can be used to track different samples and will increase the multiplexing capacities of the microfluidic devices. Samples from different sources can be placed in a known sequence such that tracking of them is facilitated, without any need of additives such as dyes; and if needed they can be merged controllably so that concentration of merged samples are the same.

There are three major types of droplet generation geometries; namely flow-focusing, co-flowing and T-junction. Owing to the simple geometry and greater ability to control the droplet size, the T-junction device is the most commonly used geometry. Zheng *et al*. used double T-junction geometry to generate droplets from two separate sources in an alternating manner^10^. This was later referred as cross junction device which has two dispersed phase inlets from opposite sides and one continuous phase inlet in the middle. None of these channels were tapered. In this work, they have observed four regimes of formation and they characterized these regimes with the capillary number, Ca, which is the ratio of viscous forces to surface tension forces. They have obtained alternating droplet formation for the range 0.001 < Ca < 0.05. They reported that for low Ca, the two dispersed phase coalescence at the junction and alternative formation does not occur. For higher Ca numbers they observed that either droplets do not form or droplets having diameters smaller than the channel width form which later merge.

Hung *et al*. developed a device to carry out a chemical synthesis for nanoparticle synthesis by first generating droplets from the two sources and then merging them at a later stage with equal or different concentrations^7^. In this work they have used a double cross junction geometry where the two dispersed phases enter the main channel from opposite sides as in Zhang *et al*.^10^. At the merging point of the dispersed channel inlets, they have used a short triangular structure named as ‘wing’ to reduce flow instabilities and backflow. They claimed that the alternating droplet formation is due to the push-pull mechanism between two dispersed phases. When one dispersed phase enters the channel, the other one is pressurized and pushed back; once a droplet is generated the second dispersed phase takes turn and pushes back the initial one. However their study did not characterize the conditions for which alternating droplet formation takes place. Nisisako *et al*. utilized the cross-junction geometry to generate core-in-shell droplets of two immiscible organic fluids, each being pumped side by side from two dispersed phase inlets^11^. Hirama *et al*. reported a device containing cross junction without taper followed by flow-focusing to encapsulate two particles, each from a different source, inside a single bigger droplet by varying the hydrophobicity inside the microchannel network^12^. This work did not focus on the mechanism of alternating droplet formation. Surya *et al*. conducted both experimental and numerical studies to identify regimes of droplet generation which were; merged, stable, transitional and laminar^13^ and used a cross junction geometry composed of straight channels to generate alternating droplets. Similar to Hung *et al*.^7^ they also explained the alternative droplet formation with the push-pull mechanism and they characterized the required Ca number and flow ratios for this mechanism, Another numerical study focusing on the same features of droplet generation but by varying the angle at which side channels merge to the main channel and viscosity ratio of the dispersed and continuous phases was performed by Ngo *et al*.^14^. They also used cross junction geometry composed of straight channels, however they changed the merging angle of dispersed phase channels and concluded that a merging angle of 90° is better for alternating droplet generation. Jin *et al*. studied the regularity of alternating mode to conclude that regularly alternating mode of droplet generation is always accompanied by regular merging of droplets^15^, but they did not study the relevance of the channel geometry to regular alternation, which further emphasizes the need for such a study that we have conducted.

**Figure 1:**
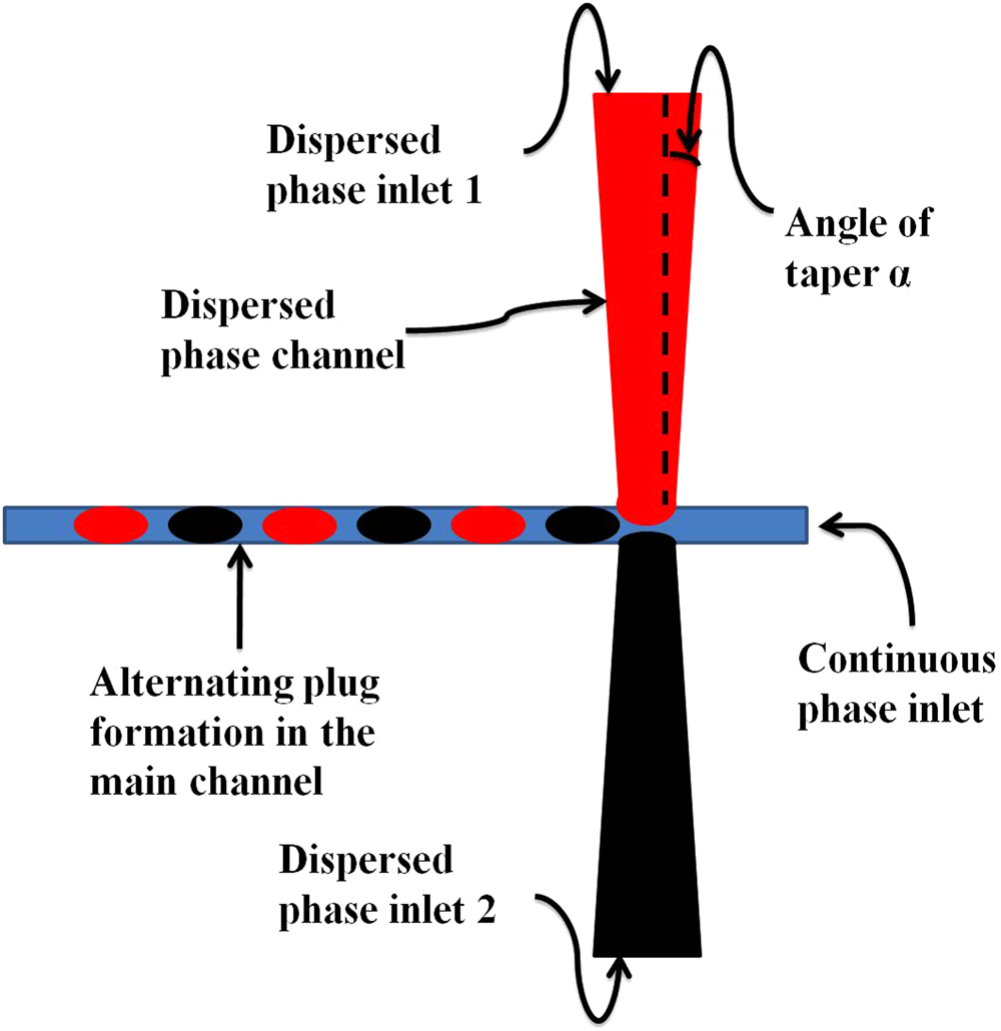
Schematic of the droplet generation device. The continuous phase inlet and the two dispersed phase inlets are shown. The angle of taper is denoted by α. Due to the presence of two dispersed phase inlets, sequential alternating droplet generation takes place. The range of values of α tested in this study is 0 ≤ α ≤ 25 in steps of 5 degrees.

This is the first study that shows how inlet channel geometry can be modified to achieve a stable alternating droplet pattern. In this work we have studied the effect of the taper angle of inlet channels (α) (Fig. 1) on the ability to generate a repeatable and stable droplet pattern. We have also defined an efficiency factor that describes how stable is the pattern formation. If the pattern is perfectly followed then such a device will be reliable in chemical synthesis due to its ability to have correct ratio of concentrations of reactants inside each microreactor and to be able to predict the contents of droplets from two different sources. Furthermore, for such a device to be compatible with automation it has to be consistent in terms of the size of droplets and the spacing between them; therefore we also explore the effect of the angle of taper on this aspect of alternating droplet generation.

## Results

### Design of the microfluidic device

In the design of the microfluidic device a cross-junction geometry is used; inlet channels are tapered instead of being straight as shown in Fig. 1. Since the alternation of droplets depends on the push pull mechanism^7^, we proposed that varying cross-sectional area of the dispersed phase inlet will affect the pressure exerted by the dispersed phase at the junction; thereby affecting the control over the sequence of droplet formation. This is the first time tapered channels have been used for the dispersed phase channels in a cross junction device for alternating droplet generation, and prior to this work each study conducted on such a device had straight channels with no variation in the cross-sectional area throughout the length of the channel. The only exception that needs to be mentioned is in the case of Hung *et al*.^7^ where they have used triangular wings to minimize flow instability and avoid back flow of reagents and they have not varied the angle of the triangular inlet to study its effect on the droplet generation.

### Additional Variables for Data Analysis

**Figure 2:**
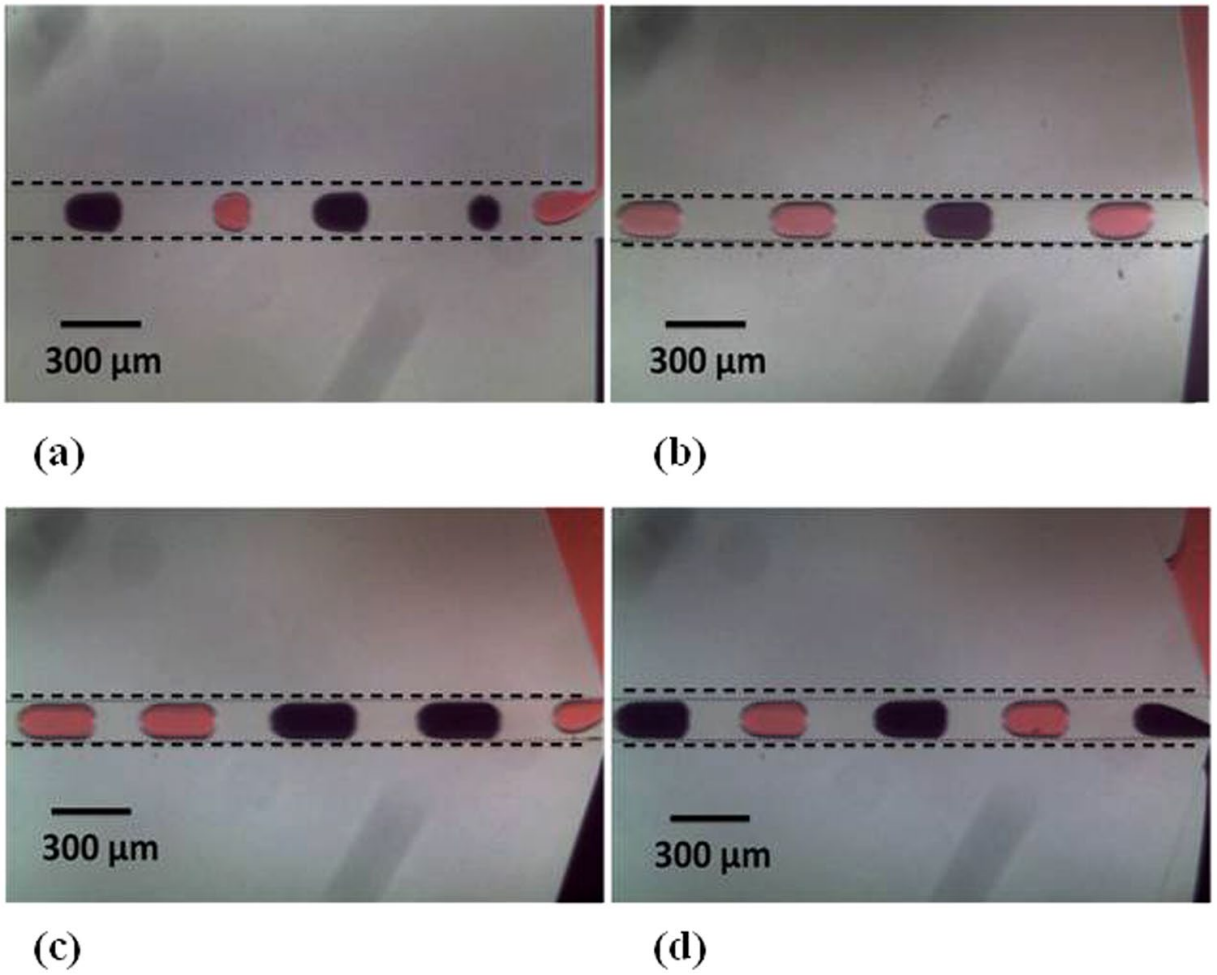
Different aspects of alternating droplet generation. (**a**) Failed attempt in which droplet dimensions are not uniform (*α* = 0°). (**b**) Failed generation attempt; number of black and red droplets are unequal (*α* = 15°). (**c**) Alternating droplet generation with *n* = 2 (*α* = 25°). (**d**) Alternating droplet generation with *n* = 1 (*α* = 25°). (Please refer to supplementary video S1).

To quantify the results of our experiments, we introduced variables n and ψ, where n is the number of consecutive droplets formed by each stream of fluid and efficiency (ψ) is defined as the number of times out of 100 generation attempts that each stream generates exactly ‘n’ droplets. If in any of the attempts, number of droplets does not equal to n or the droplet dimensions vary, it is subtracted from 100 (two types of failed generation attempts are shown in Fig. 2a,b).

### Observations for different values of α

For α = 0°, the droplet size and spacing was seen to vary during experiments (Fig. 2a). On the other hand, although the droplet size and spacing was uniform for α > 0° (as shown in Fig. 2c,d), the sequence was disrupted occasionally (Fig. 2b). This is mainly because at lower angles of taper the change of radius of curvature is lower; which has less distinguished effect in pattern generation.

**Figure 3:**
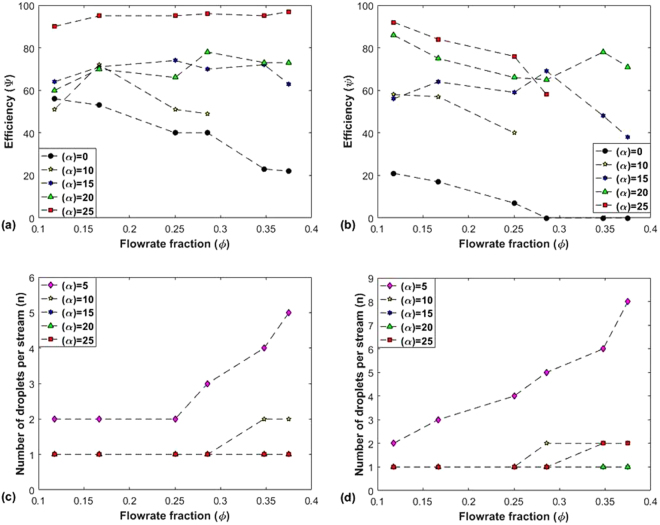
Efficiency (ψ) and number of consecutive droplets per stream (n) plotted against Flowrate fraction (*ϕ*) for two values of continuous phase flow rate (Q_c_). (**a**) Graph of efficiency (ψ) vs. flow rate fraction (*ϕ*) for n = 1 with Q_c_ = 2.0 µl/min. (**b**) Graph of ψ vs *ϕ* for n = 1 with Q_c_ = 3.0 µl/min. (**c**) Graph of number of consecutive droplets (n) vs. flow rate fraction (*ϕ*) with Q_c_ = 2.0 µl/min (**d**) Graph of n vs. *ϕ* with Q_c_ = 3.0 µl/min.

Figure 3a,b show the variation of efficiency (ψ) with ϕ. For α = 0°, ψ is the lowest as there is an absence of repeating pattern and a variation in droplet size exists. Moreover it can also be seen that ψ increases as the value of α increases. For α = 25°, the device has the greatest ability to regenerate pattern regularly with n = 1.

### Number of consecutive droplets per stream for different values of α

Furthermore it was also observed that droplet sequence having more than one alternation (Fig. 2c) could also be generated and it is expressed in Fig. 3c,d where the variation of n with ϕ for each value of α is shown. The reason for this lies in the fact that the transition from a small value of α to a higher value of α does increase the stability of droplet generation process, since the alternation transitions from no pattern in case of α = 0° to a pattern showing n > 1 in case of α = 5°, 10°. But this transition is not enough to provide complete stability for synchronized alternating droplet generation. Therefore we see that as the value of α is increased further (α = 15°), the value of n drops to 1 (as shown in Fig. 3a,b) but with a low efficiency (Fig. 3c,d). Moreover when α is 20°, the value of n remains 1, which means alternating droplet generation has reached some stability but still the efficiency is low (even though it is higher compared to the case when α = 15°). Finally the highest efficiency is obtained when α = 25° which shows that out of all the devices, our device with α = 25° is the most stable device for alternating droplet generation.

## Discussion

In order to understand the effect of the taper angle on alternating droplet generation it is necessary to look at the forces that dominate droplet formation at the T-junction. Based on the analysis proposed by Garstecki *et al*.^16^, there are three forces that act on the formation of the droplet for low Ca numbers (Ca < 10^−2^): Surface tension, forces due to shear stress applied by the continuous phase and force due to the increased resistance of fluid flow around the forming droplet. The surface tension force is a result of Laplace pressure which is the pressure difference across an interface and it depends on the radius of curvature of the interface.

In order to determine what forces are dominating the break-up, we calculated the capillary number (Ca) for our experiments and found that it is in the order of 10^−3^ as shown below:

1 $$Ca=\frac{\mu U}{\gamma }=7.94\times {10}^{-3}$$

Where the values of viscosity (*μ*), speed (*U*), cross-sectional area (A), continuous phase flow rate (Q_c_) and surface tension (*γ*) are as follows:*μ* = 0.0562 *Ns*/*m*^2^^17^, *A* = 1.49 × 10^−8^ *m*^3^/*s*, *Q*_*c*_ = 5.01 × 10^−11^ *m*^3^/*s*, *U* = 2.32 × 10^−3^ *m*/*s*, *γ* = 1.642 × 10^−2^ *N*/*m*^18^.

Based on the Ca number range droplet generation is in the squeezing regime; which means that interfacial forces are more significant than shear forces. This in turn means that droplet generation is governed by pressure distribution over the droplet^19^. When the net pressure exerted by the dispersed phase becomes smaller than the pressure exerted in the upper stream (due to continuous phase); a droplet will form^16^. The net pressure exerted by the dispersed phase is the difference of the pressure in the dispersed phase inlet channel and the Laplace pressure at the interface. Therefore by evaluating the Laplace pressure, we can determine how pressure distribution is affected at the junction.

**Figure 4:**
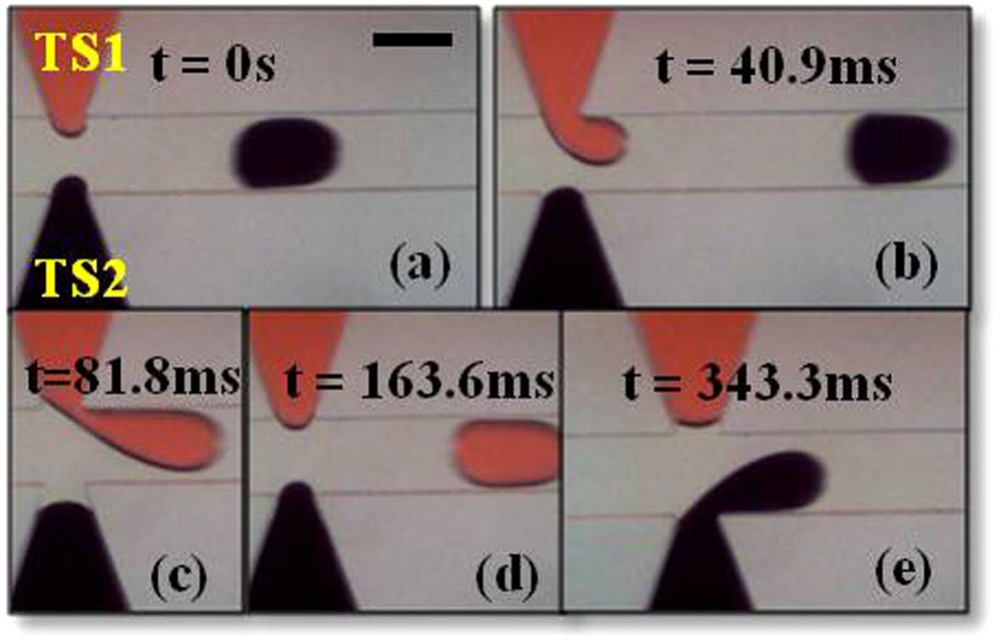
Droplet generation sequence in case of α = 25 at Q_c_ = 2.0 µl/min and Q_d_ = 0.2 µl/min. (**a**) Red stream enters the junction. (**b**) Red stream grows inside the channel slowly starting to block the junction while the black stream is pushed back by a small distance. (**c**) Red stream almost completely blocks the junction while the black stream is pushed further back. (**d**) Red stream recedes into the side channel after forming a droplet while the black stream approaches the junction. (**e**) Black stream grows inside the channel slowly starting to block the junction while the red stream is pushed back. (Scale bar: 160 µm). (Please refer to supplementary video S2).

In Fig. 4a TS1 (tapered stream 1) enters the junction as the pressure builds up. The total pressure change at the junction due to TS1 at this point is equal to^20^

2 $${P}_{TT1}={P}_{S}+{\rm{\Delta }}{P}_{TS1}$$

3 $${\rm{\Delta }}{P}_{TS1}=\frac{1}{\gamma }(\frac{1}{{R}_{H}}+\frac{1}{{r}_{H}}-\frac{1}{{R}_{T}}-\frac{1}{{r}_{T}})$$

where

- P_TT1_ is the total pressure change across the junction due to TS1
- P_S_ is the steady pressure change at the junction
- ∆P_TS1_ is the Laplace pressure difference between the head and tail interfaces of the growing droplet of TS1
- R_T_ and r_T_ are the tail radii in the axial and radial directions respectively
- R_H_ and r_H_ are the head radii in the axial and radial directions respectively.

In Fig. 4b the radius of curvature of TS1 increases therefore the value of ∆P_TS1_ decreases which shows that P_TT1_ decreases as per equation (2) and as it can be seen the TS2 is pushed slightly back (Fig. 4c) since the pressure at the junction rises. At this moment we must also realize that because of this pushing back the value of ∆P_TS2_ (Laplace pressure difference between the head and tail interfaces of the growing droplet of TS2) decreases as per equation (3) since in this case also the radius of curvature increases. For two reasons the TS2 is unable to enter the junction and is pushed back:

i. Firstly the increase in radius is small as compared to increase in radius of TS1 as it keeps filling the junction.
ii. Secondly as the TS1 keeps growing at the junction (Fig. 4c), the carrier fluid is blocked and has a limited space to flow downstream which causes an increase in the value of P_c_ (pressure in the continuous phase at the junction) and this causes an additional resistance for the TS2.

For these reasons TS2 is unable to enter the junction until droplet forms in the channel (Fig. 4c). Between Fig. 4c,d, the radial curvature of TS1 tends to go to infinity and then back to its original value^16^, and the value of this maximum pressure jump is given by^19^

4 $${\rm{\Delta }}{P}_{Lmax}=\gamma (\frac{1}{{R}_{Tc}}-\frac{1}{{R}_{Hc}})$$

where

**Figure 5:**
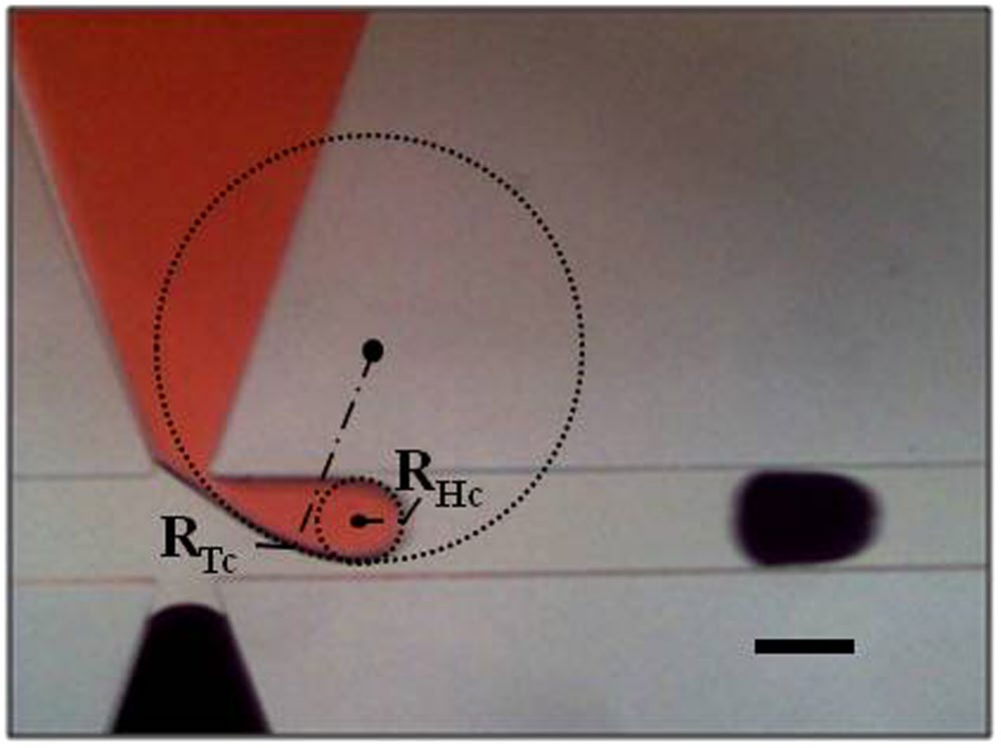
Illustration of Tail and Head Radius of curvature at break off. The radii of curvature values at the instant just before break off, R_Tc_ & R_Hc_, at the tail and head respectively. (Scale bar: 160 µm).
- ∆P_Lmax_ is Laplace pressure difference between the head and tail interfaces of the growing droplet of TS1 just before break off.
  As soon as the droplet is detached from TS1, (Fig. 4d) the junction is suddenly unblocked and the value of P_c_ decreases since carrier fluid now has the path to flow freely. As for TS2, until Fig. 4c the pressure increases and it cannot enter the junction. After the droplet of TS1 forms, due to the reduced resistance from carrier fluid and TS1, it will be able to enter the junction as shown in Fig. 4e. Accordingly in Fig. 4e it can be seen that TS1 is pushed back and the cycle repeats over and over again.
  In Fig. 6, we can see the generation of a droplet pair in the case of straight dispersed phase channels. In this case as well we define the pressure terms as earlier:
  5 $${P}_{ST1}={P}_{S}+{\rm{\Delta }}{P}_{SS1}$$
  where

- R_Tc_ and R_Hc_ are the tail and head curvature radii in the axial direction at the break off point respectively (as shown in Fig. 5).

**Figure 6:**
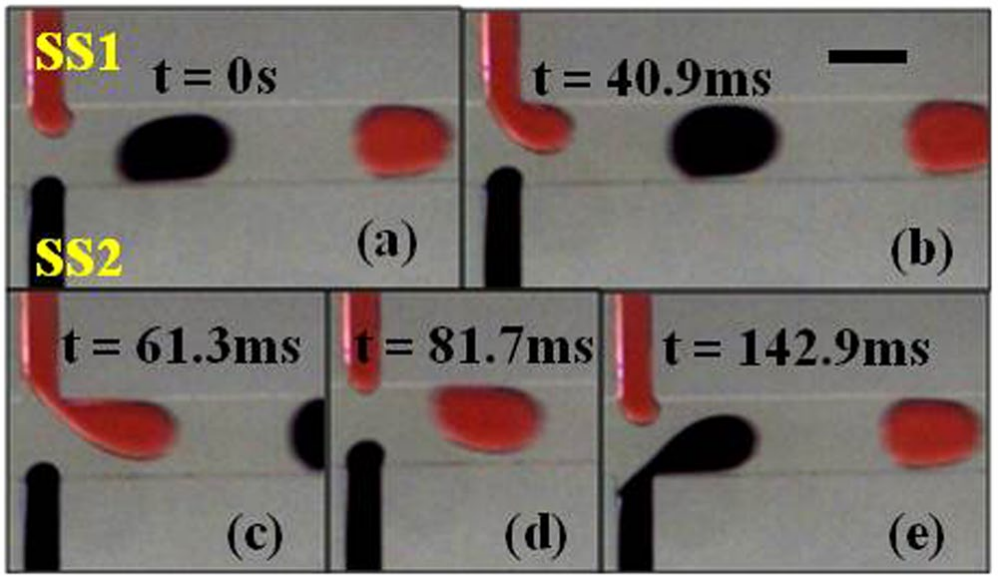
Alternating droplet generation sequence in case of α = 0 at Q_c_ = 2.0 µl/min and Q_d_ = 0.2 µl/min. (**a**) Red stream enters the junction. (**b**) Red stream grows inside the channel slowly starting to block the junction while the black stream stays at the junction. (**c**) Red stream almost completely blocks the junction while the black stream is still at the junction. (**d**) Red stream stays at the junction after forming a droplet while the black stream enters the junction. (**e**) Black stream grows inside the channel slowly starting to block the junction while the red stream also enters the junction creating a non-uniformity in alternation. (Scale bar: 160 µm). (Please refer to supplementary video S3).
- P_ST1_ is the total pressure drop across the junction due to SS1.
- ∆P_SS1_ is Laplace pressure difference between the head and tail interfaces of the growing droplet of SS1 (straight stream).

The mechanism of alternating droplet generation in case of straight channels will be partially the same as has been explained in case of tapered channels. However for the following reasons, tapered channels are more likely to produce synchronized alternating droplet pairs as compared to devices with straight channels:

i. Firstly, as shown in Fig. 4c, the TS2 is pushed back into the channel and due to the tapered geometry the radius of curvature increases which in turn means that ∆P_TS2_ decreases as per equation (3) and TS2 is more likely to enter the junction once TS1 forms a droplet. Whereas in case of straight channel geometry, as shown in Fig. 6b,c, the radius of curvature does not change since the cross section does not change. Hence in this case ∆P_SS1_ and P_ST1_ will not change; therefore SS2 is less likely to enter the junction once SS1 has formed a droplet.
ii. Secondly, the alternation of droplets depends on the second stream being able to enter the junction as shown in Fig. 6d,e. Therefore the radii of curvature values at the instant just before break off (R_Tc_ & R_Hc_) become crucial to the phenomenon of alternation.

**Figure 7:**
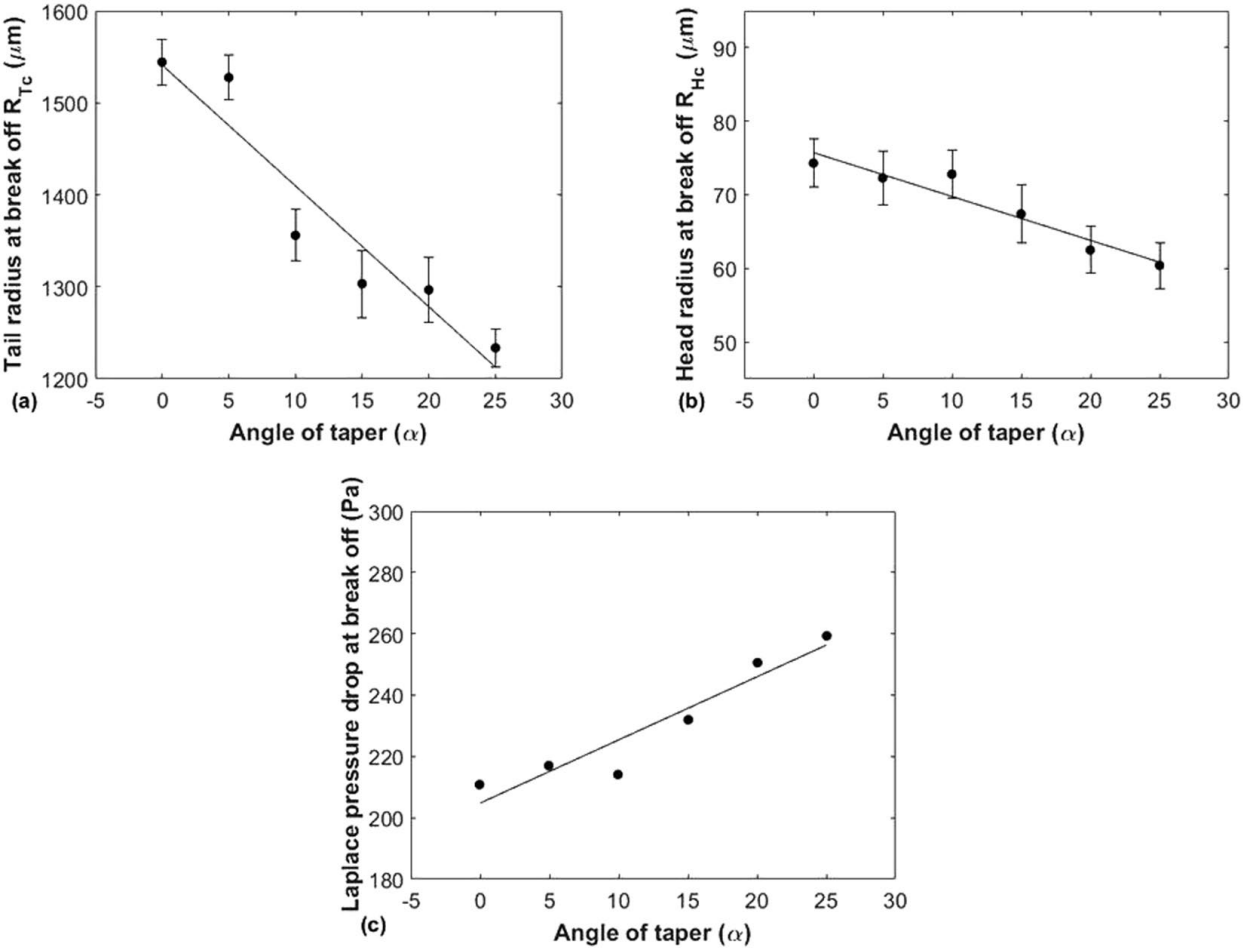
The radii in the Tail and Head directions as well as the net Laplace pressure drop plotted against α at Q_c_ = 2.0 µl/min and Q_d_ = 0.2 µl/min. (**a**) Graph of measured tail radius of curvature instantaneously before break off vs. angle of taper α, (**b**) Graph of measured head radius of curvature instantaneously before break off vs. angle of taper α, & (**c**) Graph of Laplace pressure drop at break off vs. angle of taper α.

In this work the values of R_Tc_ and R_Hc_ are measured (Fig. 5) and have been plotted in Fig. 7a,b for the flow rate combination: Q_c_ = 2.0 µl/min and Q_d_ = 0.2 µl/min to identify the effect of radius of curvature on pressure change. For each value of α, R_Tc_ and R_Hc_ are calculated separately by taking the average of radius of curvature of ten successive droplets instantaneously before break off. This means that since the data belongs to a single group, it is most appropriate to use either *range* or *standard deviation* error bars. The standard deviation error bars have been included since they are more likely to include all the values that may occur^21^.

The values of R_Tc_ and R_Hc_ are lower for tapered channels than it is for straight channels. The values of ∆P_Lmax_ were calculated using the average values of R_Tc_ and R_Hc_ in equation (3) and plotted as shown in Fig. 7c. It can be seen from this figure that ∆P_Lmax_ is higher in case of tapered channels as compared to straight channels, which according to equations (2) and (5) means that total pressure drop at the break off of first droplet will be higher for tapered channels. This will allow the TS2 to enter the junction with less resistance than in case of SS2.

At this point it must be pointed out that in straight channels, even after it forms a droplet, SS1 can still enter the junction when SS2 is generating a droplet (Fig. 6e). On the other hand, in case of tapered channels, as shown in Fig. 4e, TS1 moves back into the side channel while TS2 is generating a droplet. This points that there is a distinct pressure difference between the two streams in case of tapered geometry due to which while one of them is generating a droplet while the other stream is pushed back into the side channel. In the case of straight channels, the pressure in dispersed phases is higher and this causes random behaviors time to time as both of them can enter the channel simultaneously.

**Figure 8:**
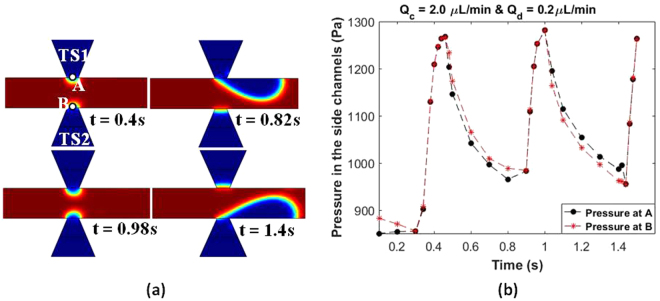
Simulation results for α = 25° at Q_c_ = 2.0 µl/min and Q_d_ = 0.2 µl/min. (**a**) Surface plot showing the generation of one droplet pair (**b**) Graph showing variation of pressure at points A and B over the droplet generation time period.

To verify this we performed simulations using *COMSOL Multiphysics ®* and the results are shown in Fig. 8. The evolution of the interface in case of one droplet pair generation is shown in Fig. 8a. The pressure variation at points A and B was plotted and is shown in Fig. 8b. At time t = 0.46 s, both streams compete to enter the junction but since the pressure at A (1267.4 Pa) is greater than the pressure at B (1266.8 Pa), TS1 enters the junction. As TS1 grows inside the junction TS2 is pushed back (Fig. 8a) due to the fact that during this time the pressure at B is higher than pressure at A. Also instantaneously before break-up, the pressure at A is 983.25 Pa (at t = 0.82 s), from which we can find *P*_*TT*1_ (equation (1)) which comes out as 284.15 Pa. From Fig. 7c we can see that the ${\rm{\Delta }}{P}_{TS1}$in case of α = 25° is 259.1 Pa. So the remaining pressure drop as per (equation (1)) is the steady pressure drop *P*_*S*_ which can be assumed to be the same in case of α = 25° and α = 0°. So we can clearly observe that *P*_*TT*1_is greater in case of α = 25° than in case of α = 0°, since the ${\rm{\Delta }}{P}_{SS1}$from Fig. 7c is 210.7 Pa. This means there is more pressure accumulation at the junction in case of α = 0° than in case of α = 25°. This can be explained by also referring to the hydraulic resistance *R*_*Hyd*_ of a rectangular channel:

6 $${R}_{Hyd}=\frac{12\mu L}{w{h}^{3}(1-0.63\frac{h}{w})}\,$$

where L is the length of the channel, w is the width of the channel, and h is the height of the channel.

All the parameters are same in case of α = 0° and α = 25°, only difference between the two geometries is the width. For α = 25° the width is the average of the two extreme widths which comes out to be 3750 µm and for α = 0° the width is uniform and the value is 60 µm. It is clear from the values of w in both cases that *R*_*Hyd*_ in case of α = 25° will be much lower than that in case of α = 0°. This will cause both streams to enter the junction at the same time in case of straight channels because of the high resistance it faces in retreating back into the main channel, thus the droplet generation will be random and unsynchronized. While in case of α = 25°, the two streams can retreat alternatively into their respective channel with greater ease and thus generate droplets in a synchronized manner.

## Methods

### Fabrication of the devices

The devices were fabricated in PDMS (Sylgard silicone elastomer, Dow Corning GmbH, Germany) by using the conventional soft lithography techniques. PDMS was bonded to a glass slide by using oxygen plasma. Syringe pumps were used to inject fluids into the microchannels. The images were captured using an inverted microscope and a CCD camera.

The continuous phase fluid was olive oil and the droplet phase fluids were deionized water with added dyes (fountain pen ink) for distinguishing the two droplet phases. The use of surfactant was avoided to understand the capability of the device without any stabilization to droplet interfaces.

### Experiment sets

The independent variables are defined as the taper angle (α), continuous phase flow rate (Q_c_) and flow rate fraction (ϕ) which is defined in equation (7)

7 $$\varphi =\frac{{{\rm{Q}}}_{{\rm{d}}1}+{{\rm{Q}}}_{{\rm{d}}2}}{{{\rm{Q}}}_{{\rm{c}}}+{{\rm{Q}}}_{{\rm{d}}1}+{{\rm{Q}}}_{{\rm{d}}2}}$$

where Q_d1_ and Q_d2_ are the flow rates of two dispersed phases.

Two sets of experiments were performed (Table 1); in set 1 the continuous phase flowrate (Q_c_) was kept at 2.0 µl/min and in set 2 it was kept at 3.0 µl/min. Within each experiment set, the dispersed phase flow rate (Q_d_) was varied but kept equal for the two dispersed phase sources, thus the ratio ϕ was varied.

**Table 1:** Testing matrices showing the combination of flow rates used in the experiments for both tapered and straight channels.

**Case#**	**Q**_**c**_ **(µl/min)**	**Q**_**d1**_ **(µl/min)**	**Q**_**d2**_ **(µl/min)**	**Flow rate fraction (ϕ)**	**Case #**	**Q**_**c**_ **(µl/min)**	**Q**_**d1**_ **(µl/min)**	**Q**_**d2**_ **(µl/min)**	**Flow rate fraction (ϕ)**
1	2.0	0.15	0.15	0.13	7	3.0	0.20	0.20	0.12
2		0.20	0.20	0.17	8		0.30	0.30	0.17
3		0.30	0.30	0.23	9		0.50	0.50	0.25
4		0.40	0.40	0.29	10		0.60	0.60	0.29
5		0.50	0.50	0.33	11		0.80	0.80	0.35
6		0.60	0.60	0.38	12		0.90	0.90	0.38

### Data Availability

The datasets generated during and/or analyzed during the current study are available from the corresponding author on reasonable request.

## Electronic supplementary material

Supplementary Video S1

Supplementary Video S2

Supplementary Video S3
